# Preservation of the Antioxidant Capacity of Resveratrol via Encapsulation in Niosomes

**DOI:** 10.3390/foods10050988

**Published:** 2021-04-30

**Authors:** Noelia D. Machado, Gemma Gutiérrez, María Matos, Mariana A. Fernández

**Affiliations:** 1 Facultad de Ciencias Químicas, Departamento de Química Orgánica, Universidad Nacional de Córdoba, Ciudad Universitaria, Córdoba X5000HUA, Argentina; nmachado@fcq.unc.edu.ar; 2 Instituto de Investigaciones en Físico-Química de Córdoba, INFIQC-CONICET, Córdoba X5000HUA, Argentina; 3 Departamento de Ingeniería Química y Tecnología del Medio Ambiente, Universidad de Oviedo, Julián Clavería 8, 33006 Oviedo, Spain; gutierrezgemma@uniovi.es (G.G.); matosmaria@uniovi.es (M.M.); 4 Instituto Universitario de Biotecnología de Asturias, University of Oviedo, 33006 Oviedo, Spain

**Keywords:** niosomes, phytoalexin, resveratrol, antioxidant activity, photoprotection

## Abstract

Resveratrol (RSV) is a natural polyphenol which produces several benefits to human health, being the *trans*-isomer the most bioactive. However, its systemic absorption is limited due to its low water solubility, that reduces the oral bioavailability, and its chemical instability (owing to the *trans*-*cis* RSV isomer conversion upon light irradiation). Thus, encapsulation of this bioactive compound is required to protect it from destructive environmental conditions. Here, *trans*-RSV was encapsulated in food grade nanovesicles formed by Tween 80 and Span 80, with or without the addition of dodecanol (Dod) as membrane stabilizer. The size and shape of niosomes were evaluated by microscopy (TEM) and light scattering. RSV was successfully encapsulated in the vesicular systems (49–57%). The effect of Dod in the membrane bilayer was evaluated on the RSV in vitro release experiments under simulated gastrointestinal conditions. The total antioxidant capacity of the encapsulated polyphenol was measured using radicals’ assays (DPPH and ABTS). The niosomes were able to maintain almost the total antioxidant capacity of encapsulated RSV, also preserved the ~85% of *trans*-RSV, thus offering considerable protection against high energy irradiation. These results make these systems suitable for different applications, particularly for photosensitive compounds.

## 1. Introduction

Resveratrol (RSV, trans-3,5,4’-trihydroxystilbene) is a polyphenol found in natural sources such as grapes, soy beans, berries, nuts and pomegranates [1]. The *trans*-isomer of RSV is more bioactive than the *cis*-isomer and besides, more abundant in Nature. It is a secondary metabolite produced by plants in response to biotic and abiotic stress [2]. The interest of the scientific community in this compound relies in its benefits for human health. It has been proved that RSV has antioxidant [3], anti-inflammatory [4], and anti-carcinogenic activity [5], as well as cardioprotection [6], among others [7,8].

Although several foods contain this compound, its systemic absorption in the human body is limited, and only less than 1% of consumed RSV is finally absorbed [9]. The factors which restrict its efficacy are the low water solubility (<0.05 mg/mL), the reduced oral bioavailability [10] and their chemical instability. The low oral bioavailability of RSV is result of the sulfation and glucuronidation reactions in the phase I and II of the metabolism in the gastrointestinal tract [5]. Regarding the chemical instability, *trans*-RSV isomer could be transformed to *cis* isomer after light irradiation. In fact, approximately 80–90% of *trans* isomer could be isomerized when it is exposed to sunlight, high intensity white light, or UV light of 360 nm and 264 nm [11,12]. Besides, its chemical degradation is not only caused by the light exposure but also by high temperatures [13], pH changes [13,14], and exposure to different types of enzymes [15,16]. As a consequence, it might be susceptible to degradation by food processing operations, adverse environmental conditions, and gastrointestinal digestion [17]. Thus, encapsulation of this bioactive compound is required to protect it from unfavorable environmental circumstances.

Several delivery systems have been developed to control retention, stability, and release of bioactive compounds [18], for example, microemulsions [19], simple [20] and double emulsions [20,21], particles [22,23], gels [24], nanocapsules [25], nanocrystals [26], and vesicles [27,28]. Particularly, vesicles show special possibilities as bioactive compounds carriers, due to their unique properties such as similarity with biomembranes, high surface-volume ratio, and ease of drug-release modulation [29].

Niosomes are vesicular systems formed when non-ionic surfactants are exposed in an aqueous medium. They are highly organized and they consist of concentric hydrophobic bilayers that enclose an inner aqueous compartment. Niosomes offer a variety of advantages over other vesicular systems, such as versatility, biocompatibility, chemical stability, low cost, and in addition they can be easily derivatized [29,30]. Due to these characteristics, niosomes present a wide range of potential applications in the food industry.

In niosomes, as occurs with liposomes, water soluble active molecules are preferably located in the internal aqueous core compartment, whereas the lipophilic ones remain entrapped within the lipidic bilayer. The further release of the active compound from vesicles depends, among other factors, on the bilayer composition [31]. It was found that, when cholesterol was used as membrane stabilizer, it altered bilayer permeability [32]. Moreover, some fatty alcohols were also used as bilayer stabilizers, finding that the release of solutes from these niosomes could be optimized by altering membrane constituents and their concentrations [31]. In a previous work, loaded RSV niosomes were prepared using dodecanol (Dod) as membrane stabilizer, while Span 60 or Maisine 35-1 were used as main membrane compounds [33]. It was verified that Dod could replace cholesterol as stabilizer in food-grade formulations of niosomes.

In most studies, the encapsulation efficiency values of the formulated colloidal systems are a key parameter to be optimized for further applications as part of nutraceutical or cosmeceutical products (with both cosmetic and therapeutic effects). However, in addition to the encapsulation efficiency, it is also of great interest to know the total antioxidant capacity of the encapsulated RSV, considering the protection against photoisomerization of the *trans*-RSV, since it will be a determining factor for the potential benefits of the final product. There is a general consensus that the future for efficient resveratrol delivery is based on obtaining systems capable of overcoming the limitations of this compound. There are a few reports based on the use of different delivery systems for the photoprotection of RSV, such as, self-nanoemulsions, core-shell nanoparticles, cyclodextrin complexes, hydrogels and lipid-core nanocapsules [18,34,35]. For instance, Fan et al. developed RSV-loaded zein encapsulated with bovine serum albumin-caffeic acid conjugated core-shell nanoparticles. RSV encapsulated showed greater stability against both heat and ultraviolet light treatments, without loss of antioxidant activity [36]. In other study, essential oil based microemulsions for topical applications significantly improved the solubility of RSV maintaining a high free radical scavenging activity [37]. However, the employment of nanovesicles formulated with non-ionic surfactants for photoprotection of resveratrol was scarcely studied.

Therefore, looking for a liquid formulation to stabilize RSV, protect it from degradation, improve its water dispersibility, and maintain its antioxidant activity, in the present work *trans*-RSV was encapsulated in niosomes formulated by mixtures of Tween 80 (Tw80) and Span 80 (Sp80) [38], using dodecanol (Dod) as membrane stabilizer. The effect of Dod in the membrane bilayer was evaluated on the RSV in vitro release experiments from the niosomes. Moreover, the protection offered by the vesicles to the photoisomerization of *trans*-RSV, as well as the ability to maintain the total antioxidant capacity of the encapsulated polyphenol, was evaluated for the different formulations used. The main goal of this work was to find a suitable system with high encapsulation efficiency and that also offering sufficient protection to *trans*-resveratrol against photoisomerization.

## 2. Materials and Methods

### 2.1. Materials

Tw80 was purchased to Riedel-de Haën (Munich, Germany) and Sp80 to Fluka (Buchs, Switzerland). Dod, hydrochloric acid, potassium chloride, sodium hydroxide, potassium phosphate monobasic and potassium persulfate were supplied by Merck (Darmstadt, Germany). 2,2’-Azinobis(3-ethylbenzthiazoline-6-sulfonic acid), (ABTS) ≥ 98%, *trans*-RSV ≥ 99%, and 2,2-diphenyl-1-picrylhydrazyl (DPPH) ≥ 98% were provided by Sigma Aldrich (St. Louis, MO, USA). Ethanol absolute and methanol HPLC quality were obtained by Sintorgan (Buenos Aires, Argentina). MilliQ water was used in all experiments.

Niosomes formed by the mixture of Tw80 and Sp80 were named Tw80:Sp80 niosomes and that composed by the mixture of Tw80, Sp80 and Dod were called Tw80:Sp80:Dod niosomes.

### 2.2. Preparation of Niosomes

The thin film hydration method was employed to prepare the niosomes. Stock solutions of Tw80 (0.153 M), Sp80 (0.237 M), Dod (0.067 M), and RSV (0.044 M) were prepared in ethanol. The solutions were stored at 4 °C and, particularly, RSV solutions were also protected from the light. As it was aforementioned, Dod is a slightly water soluble fatty alcohol that could be used as membrane stabilizer since it modifies membrane permeability as cholesterol [31,33]. In preliminary tests, the amount of Dod added was optimized by testing different surfactants:Dod ratios, in a range of 0.004–7 times of alcohol, relative to the total concentration of surfactants. The lower limit was the aqueous solubility of Dod, and the upper limit was the concentration previously used to stabilize niosomes formulated with Span 60 [33]. Finally, from the different assays, the relation Tw80:Sp80:Dod (1:1:1) was chosen to the further assays.

For Tw80:Sp80 niosomes, 0.21 mL of Tw80 and 0.33 mL of Sp80 from stock solutions were added in a 50 mL round bottom flask. For Tw80:Sp80:Dod niosomes, 0.14 mL of Tw80, 0.22 mL of Sp80 and 0.50 mL of Dod from stock solutions were added. In case of RSV loaded niosomes, 0.05 mL of RSV stock solution were added in this step. Then, ethanol was aggregated to reach 10 mL. All the organic solvent was evaporated under vacuum using a Büchi R-200 rotary evaporator (Merck, Darmstadt, Germany) to form a thin film at the bottom of the flask. After, 10 mL of water was aggregated and the new suspension was stirred at 116 strokes per minute (spm) during 30 min at 60 °C in an OLS200 Grant shaker water bath (Grant Instruments, Royston, UK). Finally, the suspension was left to equilibrate at room temperature and thus multillamellar vesicles were formed. For both vesicular systems, the final concentration of membrane components was 10 mM.

### 2.3. Niosomes Size and Morphology

Dynamic light scattering (DLS) was used to determined size and polidispersity index (PDI). The equipment used was a particle analyzer Delsa™ Nano C Beckman Coulter Inc. (Life Sciences, Indianapolis, IN, USA). The measurements were carried out with 658 nm laser at 25 °C. Standard deviations (±SD) were obtained for the average value of three diameter measurements.

Vesicles morphology was determined using transmission electron microscopy (TEM). The 1200 EX II microscope (JEOL, Tokyo, Japan) worked at an accelerating voltage of 80 kV. A solution of uranyl acetate 2% (*w/v*) was used as staining agent. A drop of niosomal suspension was placed on a carbon coated copper grid and then was left 1 min to ensure adhesion process. The excess of solution dispersion was removed using a piece of filter paper. Then, a drop of the staining agent was placed over the carbon grid and the same procedure was performed.

### 2.4. Encapsulation Efficiency 

Encapsulation efficiency (*EE*) is defined as the percentual ratio between the quantity of encapsulated RSV (remaining after purification using exclusion gel chromatography) and the total RSV added during niosome preparation (Equation (1)):

(1) $$EE=\frac{{\left[\mathrm{RSV}\right]}_{encapsulated}}{{\left[\mathrm{RSV}\right]}_{total\;added}}\times 100$$

The separation of free RSV from RSV-loaded vesicles was carried out by exclusion gel chromatography based on differences in size. This procedure allows fast separation of high molecular weight substances (RSV-vesicles) which are excluded from the matrix pores and are eluted first, from low molecular weight molecules (free RSV). According to the gravity protocol, 2.5 mL of RSV-niosomes were loaded in a Sephadex G-25 column (Desalting Columns, GE Healthcare Life Sciences, Chalfont St. Giles, UK) using water as mobile phase. The first 3.5 mL of eluate were collected and used for quantification of RSV encapsulated.

Then, 0.1 mL of purified and not purified RSV-niosomes were diluted in 1 mL of methanol and, after that, RSV concentration was determined using RP-HPLC (HP series 1100 chromatograph, Hewlett Packard, Agilent Technologies, Santa Clara, CA, USA) with a Zorbax Eclipse Plus C18 (5 μm, 4.6 mm × 150 mm column, Agilent Technologies, Santa Clara, CA, USA). The detector employed was a UV/vis (HP G1315A, Agilent Technologies, Santa Clara, CA, USA) and the λ_max_ of RSV detection was 310 nm. The RP-HPLC quantification procedure was described in previous studies [39]. Milli Q water was used as mobile phase A and methanol as mobile phase B. The gradient started with 80% of A, reaching 100% of B after 5 min, maintaining it for another 10 min. The flow rate was 0.8 mL/min and the retention time for RSV was 6.1 min.

### 2.5. In Vitro Release Experiments

Release experiments were performed under simulated gastrointestinal conditions at 37 °C using Franz diffusion cells. To achieve gastric conditions, a buffer solution at pH 1.2 was prepared with HCl/KCl, 0.085 M/0.050 M, and to imitate intestinal conditions, a buffer at pH 6.8 was prepared with NaOH/KH_2_PO_4_, 0.022 M/0.050 M, as was previously described [35]. The donor compartment of the cell was filled with RSV loaded niosomes whereas the receptor compartment was filled with the corresponding buffer solution. Both compartments were separated with a dialysis benzoylated membrane (Sigma Aldrich, cutoff 2 kDa MW). At specific time intervals (every 15 min at pH 1.2 for 2 h, and every 1 h at pH 6.8 during 6 h), 1 mL of solution was carefully removed for subsequent RP-HPLC analysis to evaluate RSV concentration. The removed volume was replaced with fresh buffer solution (1 mL). The RSV concentration was determined from a calibration curve and the cumulative RSV release was obtained by calculating the total amount detected in the aliquots.

### 2.6. Antioxidant Activity

#### 2.6.1. DPPH Test

A standard solution of DPPH (60 μM) in methanol was prepared daily and protected from light (λ_max_ 515 nm). RSV-niosomes (0.1 mL) were added to 3 mL of DPPH solution in a quartz cuvette (1 cm path length). After quickly homogenization, the absorbance at 515 nm was measured every 1.5 min until a plateau was reached, using a UV-VIS spectrophotometer UV-1800 (Shimadzu, Tokyo, Japan). A methanolic solution of RSV 3 µM was employed as control for both vesicular systems.

#### 2.6.2. ABTS Test

A standard solution of ABTS radical was prepared as follow: 38.8 mg of ABTS and 6.7 mg of potassium persulfate were placed in a 10 mL volumetric flask adding water to reach the final volume. This solution was left 12 h to ensure the radical ABTS formation. This radical presented a λ_max_ at 734 nm. RSV-niosomes (0.1 mL) were added to 3 mL of ABTS solution in a quartz cuvette (1 cm path length). After homogenization, the absorbance of the solution was measured at 734 nm each 1.5 min until no changes, using a UV-VIS spectrophotometer. An ethanolic RSV solution 4 µM was used as control for Tw80:Sp80 niosomes, and a 3 µM RSV ethanol solution as control for Tw80:Sp80:Dod niosome system.

### 2.7. Stability of Trans-RSV against Photoisomerization Induced by UV Light

Samples of RSV in Tw80:Sp80 niosomes, RSV in Tw80:Sp80:Dod niosomes and RSV in ethanolic solution were placed under type UV-C light (100–280 nm, 15 W) during 15 min. The RSV concentrations were quantified using RP-HPLC before and after the irradiation.

### 2.8. Statistical Analysis

All data were expressed as the mean ± SD (standard deviation) of three independent experiments, and statistical analysis of the data was carried out (*t*-Student) at the 95% confidence level using statistical software (InfoStat versión 2020, Universidad Nacional de Córdoba, Córdoba, Argentina).

## 3. Results and Discussion

Niosomes were formulated with Tw80:Sp80 (1:1) and Tw80:Sp80:Dod (1:1:1) as was mentioned in Section 2.2. The systems were characterized by size of the vesicles, RSV encapsulation efficiency and its in vitro release. In order to know about the functionality of the systems, antioxidant activity and stability of RSV were also analyzed. 

### 3.1. Niosomes Size and Size Distribution

The mean diameters and PDI values of the prepared niosomes are shown in Table 1. In general, RSV-niosomes had an average size of approximately 445 nm while empty niosomes presented a diameter near to 290 nm. Both systems had mean PDI values of 0.36 indicating a narrow size distribution [40].

According to Table 1, a significant increase in the size of empty niosomes was observed due to the presence of Dod on the membrane bilayer (*p*-value 0.0145 < 0.05), probably caused by differences in the curvature radius. On the other hand, the presence of RSV produced an increase in the average diameter compared to the empty niosomes for both compositions (*p*-values < 0.05), and no significant differences were found in loaded niosomes as consequence of the addition of dodecanol (*p*-value 0.3656 > 0.05). It is known that the encapsulation of different molecules can increase vesicles size without affecting their morphology, as previously observed Tavano et al. [41].

**Figure 1:**
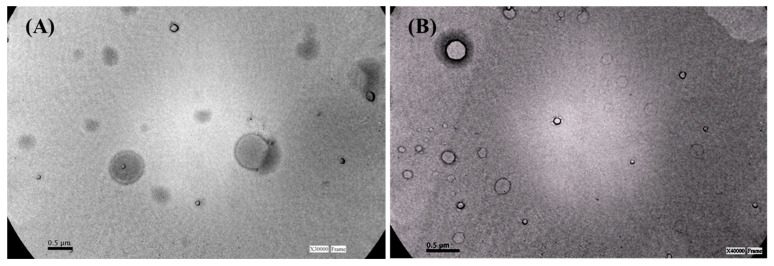
TEM photomicrographs of (**A**) RSV-Tw80:Sp80 niosomes and (**B**) RSV-Tw80:Sp80:Dod niosomes.

Figure 1 shows TEM images of RSV-loaded niosomes of Tw80:Sp80 (Figure 1A) and of Tw80:Sp80:Dod (Figure 1B). The presence of Dod did not alter the morphology of vesicles. In both cases, the niosomes were spherical, with sizes in good agreement with those determined by DLS measurements.

### 3.2. Encapsulation Efficiency

After the purification of the niosomes by size exclusion gel chromatography, the quantification of encapsulated RSV was performed. The addition of Dod did not produce significant changes in RSV encapsulation efficiency values using Tw80:Sp80 niosomes (*p*-value 0.2073 > 0.05). The values were (49 ± 9)% vs. (57 ± 2)% for niosomes without and with Dod respectively (Table 1). Besides, the reached values were close to those obtained in previous studies. For instance, Pando et al. prepared niosomes containing RSV with Span 60-Dod and glyceryl monolinoleate-Dod, obtaining an EE value of (64 ± 8)% and (53 ± 8)%, respectively [33]. However, Vankayala et al. prepared niosomes using Span 60 and cetyl alcohol as membrane stabilizer, obtaining an EE of RSV equal to (81 ± 2)%. The increase in the length of the alcohol hydrocarbon chain probably allows a better packing between amphiphilic molecules, in comparison with a short alcohol. In this sense, the entrapment of small hydrophobic molecules such as RSV it is favored via hydrophobic interactions, resulting in a higher EE [42].

Moreover, the concentration of RSV encapsulated inside niosomes (0.085 and 0.093 mM in niosomes with and without Dod) was included in the therapeutic dose according with some previous studies [43], and it is similar to that used by Pando et al. for the enrichment of yogurts [33].

### 3.3. In Vitro Release of RSV

The release of RSV from niosomes with and without Dod was measured at 37 °C at pH 1.2 (to emulate gastric conditions) and pH 6.8 (to mimic intestinal conditions). The RSV release from Tw80:Sp80 niosomes was almost complete in both media, reaching values of (86 ± 4)% at pH 1.2 during 2 h and (97 ± 3)% at pH 6.8 in 6 h. On the contrary, the RSV release at acidic pH was slower from niosomes with Dod ((47 ± 3)%) whereas at pH 6.8 was similar ((94 ± 2)%) at the same time (Table 1). 

The presence of Dod significantly modified the RSV release at pH 1.2. Considering that at pH 1.2 any of the OH groups in RSV are deionized (RSV pKa are 8.8, 9.8 and 11.4) [44], the observed difference is attributed to the presence of Dod. These results suggest that membrane permeability is affected by the fatty alcohol, which is modulating the release of RSV through it at this pH.

It is known that RSV is quickly absorbed in the upper tract of small intestine; moreover, the small size and the non-polar character of this molecule facilitate its absorption through membranes via passive diffusion [45]. The in vitro release experiments showed that RSV could be completely released from both type of niosomes in intestinal conditions. However, niosomes with Dod controlled RSV release in gastric conditions by halving it, comparing with the niosomes free of alcohol.

### 3.4. Antioxidant Activity

**Figure 2:**
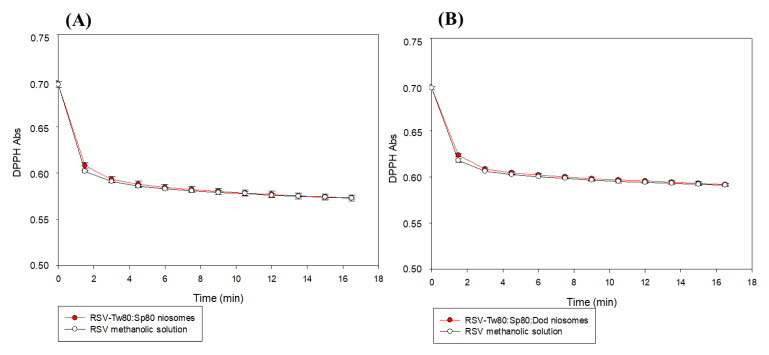
DPPH absorbance vs. time for (**A**) Tw80:Sp80 and (**B**) Tw80:Sp80:Dod niosomes, loaded with RSV, and a control solution in methanol. [RSV] = 3 μM. Error bars correspond to standard deviations of at least three determinations.

The antioxidant capacity of RSV was studied by the DPPH method and by the ABTS assay. The results obtained for RSV encapsulated in Tw80:Sp80 and in Tw80:Sp80:Dod niosomes in presence of DPPH, are shown in Figure 2. In both cases, the results were compared with a control solution of RSV in methanol at the same concentration.

It can be observed that the decay of the DDPH radical absorbance after the addition of RSV encapsulated in both type of niosomes (with and without Dod) is similar to the control (RSV-free), Table 1. These results confirm the retention of the antioxidant activity of RSV after its encapsulation in niosomes. When vesicles are exposed to an organic solvent, such as methanol, the vesicles structure is disorganized and, in consequence, RSV is released, and available to perform its antioxidant action. Similar results were observed by Vankayala et al., who reported that RSV maintained its antioxidant activity after encapsulation in Span 60 niosomes and cetyl alcohol [42].

**Figure 3:**
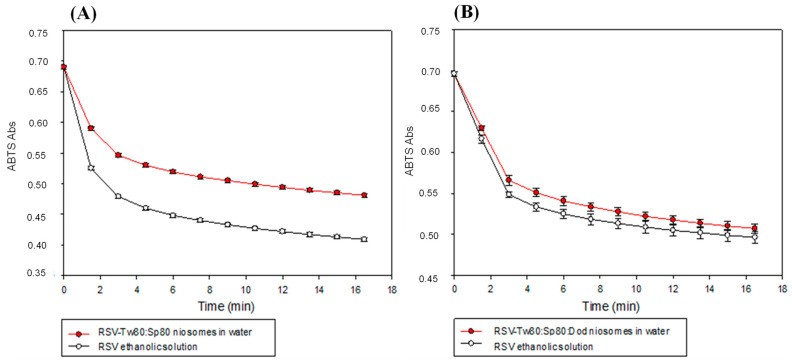
ABTS absorbance vs. time for (**A**) Tw80:Sp80 and (**B**) Tw80:Sp80:Dod niosomes, loaded with RSV, and a control solution in ethanol. (**A**) [RSV] = 4 μM, (**B**) [RSV] = 3 μM. Error bars correspond to standard deviations of at least three determinations.

The ABTS assay allows to obtain knowledge about the antioxidant capacity of RSV inside the vesicles because it can be carried out also in aqueous solutions [46]. In this test, once the ABTS radical cation has been formed (through the reaction between ABTS and potassium persulfate), RSV was added and a reduction of the radical absorbance was observed. The results obtained for RSV in Tw80:Sp80 and Tw80:Sp80:Dod niosomes in the ABTS assay are shown in Figure 3 and Table 1. For both systems, a solution of RSV in ethanol at the same concentration, was used as control. 

In this test, the antioxidant capacity of the encapsulated RSV in Tw80:Sp80 niosomes was lower than the activity in the control solution. After 16 min, the antioxidant activity of the encapsulated RSV was (75 ± 1)% of the total, compared to the RSV in the control solution (100%). This result could be due to the shielding or protective effect offered by the niosomes to RSV because of the encapsulation.

**Figure 4:**
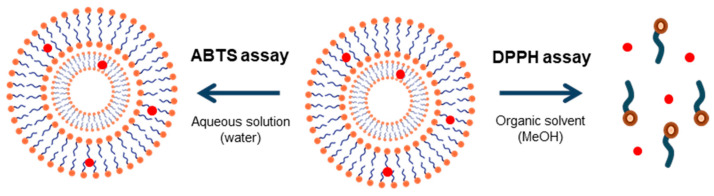
Organization of amphiphilic molecules in the antioxidant assays.

Owing to the fact this test was carried out in water, the vesicular systems was stable and not disorganized as in the case of DPPH, where RSV are free after rupture of vesicles in methanol (Figure 4). Similar results were obtained by Feng et al., who observed that the antioxidant capacity of (+)-catechin and (–)-epigallocatechinlaurate encapsulated in vesicles of dodecyl gemini O-glucoside against the radical cation ABTS, was lower in relation to the compounds in solution, attributing this result to the same effect [47].

On the other way, in the same test, it was observed that the antioxidant capacity of RSV encapsulated in Tw80:Sp80:Dod niosomes was similar to the control solution (96% vs. 100%) (Figure 3B). This result would indicate that RSV could be more exposed to the hydrophilic interface of the system when Dod is present, being available to perform its antioxidant activity. Although ABTS is not much used in water, a comparable example is the work of Doppalapudi et al., who developed ultradeformable liposomes for the co-encapsulation of psoralen and RSV for the treatment of vitiligo. In that work, the encapsulated and free RSV (from control solution) presented the same antioxidant activity against the radical cation ABTS at the same concentration [48], in a similar way to the results observed here.

### 3.5. Stability of Trans-RSV against Photoisomerization Induced by UV Light

**Figure 5:**
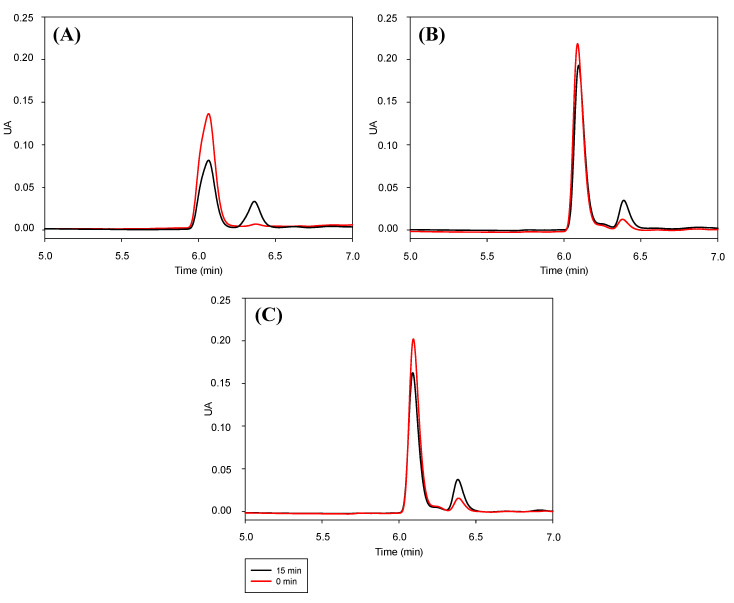
Chromatograms of (**A**) RSV ethanolic solution, (**B**) RSV-Tw80:Sp80 and (**C**) RSV-Tw80:Sp80:Dod niosomes at 0 min (red line) and 15 min (black line) after UV light exposure.

Another important characteristic to be studied is the stability of RSV encapsulated in niosomes against isomerization induced by UV light. After preparation, the formulations loaded with RSV and a solution of RSV in ethanol as control, were irradiated with UV-C. This type of light corresponds to the higher energy area of the electromagnetic spectrum. Figure 5 shows chromatograms with two peaks corresponding to *trans*-RSV (t_R_ = 6.06 min) and *cis*-RSV (t_R_ = 6.37 min). These peaks were identified based on previous published works in which the *cis* isomer of RSV has longer retention times than the *trans* isomer, under reverse phase conditions in HPLC [14,49].

After 15 min of irradiation, the intensity of the *trans*-RSV peak decreased while the *cis*-RSV increased, indicating photoisomerization. However, this decrease was different if the polyphenol was free (in the ethanolic solution) or encapsulated in niosomes. Thus, the concentration of the *trans* isomer in the ethanolic solution decreased (42 ± 3)% while only the (13 ± 4)% and (19 ± 4)% of the *trans*-RSV was reduced in Tw80:Sp80 and Tw80:Sp80:Dod niosomes, respectively (Table 1). These results indicated that the presence of niosomes inhibits the photoisomerization of RSV even when high frequency radiation is used. To the best of our knowledge, this is the first analysis of the protection given by niosomes to the photoisomerization of RSV employing high-energy UV light. However, there are another works where different kind of nano carriers were used. For instance, Koga et al. encapsulated RSV in sodium caseinate microparticles. The authors irradiated the microparticles with UV light at 365 nm during 1 h. Then, the 36% of the encapsulated bioactive RSV was consumed, while 51% did so from a control solution [50]. On the other hand, Liu et al. developed particles of protein-polyphenol complexes for the co-transport of RSV and curcumin. After the exposure to UV light for 1 h, the 35% of the *trans* isomer dissolved in DMSO was photoisomerized, while only 10% of RSV encapsulated in the nanoparticles was reduced. The authors attributed this effect to the low intensity of UV light that reached the RSV as a result of the scattering or absorption of the light by the nanocarrier [51]. This last result is in concordance with the protection offered to *trans* RSV by niosomes in this work. It is important to mention that the inclusion of Dod in the carrier did not seem to produce significative differences in the photoprotection of RSV.

## 4. Conclusions

A new food grade transport system for RSV encapsulation with interesting possibilities was proposed. In this sense, the studied niosomal formulations consisting of surfactants approved for their use in foods, free of cholesterol, presenting a size and PDI values suitable for food applications. Specifically, RSV could be encapsulated in both vesicular systems studied, and the amount of loaded RSV was similar to those obtained in other formulations already used in food matrices. In general, the presence of Dod modified the release of RSV in gastric conditions without affecting general characteristics like size, shape, and antioxidant activity. Regarding antioxidant activity, these niosomes were able to preserve the total antioxidant capacity of RSV, offering also protection against *trans*-RSV photoisomerization. To the best of our knowledge, this is the first analysis of the protection given by niosomes to the photoisomerization of RSV employing high-energy UV light.

Finally, the studied niosomal systems demonstrated to stabilize RSV, protecting it from degradation, improving its dispersibility in water and maintaining its antioxidant activity. The reported systems have significant potential as bioactive delivery vehicles in food area.

## Figures and Tables

**Table 1:** Composition, mean diameter, polydispersity index (PDI) and encapsulation efficiency (EE) values, percent of RSV released, antioxidant activity and RSV chemical stability of niosomes prepared. (±SD, *n* = 3).

Niosome Composition	Diameter (nm)	PDI	EE (%)	% RSV Released Gastric Conditions	% RSV Released Intestinal Conditions	Antioxidant Activity (%)		Chemical Stability (% *Trans*-*Cis* Conversion)
						DPPH	ABTS	
Tw80:Sp80	267 ± 10	0.37	-	-	-	-	-	-
RSV-Tw80:Sp80	469 ± 73	0.37	49 ± 9	86 ± 4	97 ± 3	100	75 ± 1	13 ± 4
Tw80:Sp80:Dod	312 ± 16	0.36	-	-	-	-	-	-
RSV-Tw80:Sp80:Dod	420 ± 40	0.34	57 ± 2	47 ± 3	94 ± 2	100	96 ± 2	19 ± 4

RSV = resveratrol; Tw80 = Tween 80; Sp80 = Span 80; Dod = dodecanol; DPPH= 2,2-diphenyl-1-picrylhydrazyl; ABTS= 2,2’-Azinobis(3-ethylbenzthiazoline-6-sulfonic acid).
